# Identification of genes escaping X inactivation by allelic expression analysis in a novel hybrid mouse model

**DOI:** 10.1016/j.dib.2015.10.033

**Published:** 2015-11-03

**Authors:** Joel B. Berletch, Wenxiu Ma, Fan Yang, Jay Shendure, William S. Noble, Christine M. Disteche, Xinxian Deng

**Affiliations:** a Department of Pathology, University of Washington, Seattle, WA, USA; b Department of Genome Sciences, University of Washington, Seattle, WA, USA; c Department of Medicine, University of Washington, Seattle, WA, USA

**Keywords:** RNA-seq, ChIP-seq, X inactivation, Transcription factor, Gene expression, Allele-specific

## Abstract

X chromosome inactivation (XCI) is a female-specific mechanism that serves to balance gene dosage between the sexes whereby one X chromosome in females is inactivated during early development. Despite this silencing, a small portion of genes escape inactivation and remain expressed from the inactive X (Xi). Little is known about the distribution of escape from XCI in different tissues in vivo and about the mechanisms that control tissue-specific differences. Using a new binomial model in conjunction with a mouse model with identifiable alleles and skewed X inactivation we are able to survey genes that escape XCI in vivo. We show that escape from X inactivation can be a common feature of some genes, whereas others escape in a tissue specific manner. Furthermore, we characterize the chromatin environment of escape genes and show that expression from the Xi correlates with factors associated with open chromatin and that CTCF co-localizes with escape genes. Here, we provide a detailed description of the experimental design and data analysis pipeline we used to assay allele-specific expression and epigenetic characteristics of genes escaping X inactivation. The data is publicly available through the GEO database under ascension numbers GSM1014171, GSE44255, and GSE59779. Interpretation and discussion of these data are included in a previously published study (Berletch et al., 2015) [1].

## Specifications

**Table:** 

Organism/cell line/tissue	*Mus musculus (C57BL/6J) x Mus spretus F1 whole brain, whole spleen, whole ovary, and Patski fibroblasts derived from embryonic kidney*

Sex	*Female*
Sequencer or array type	*Illumina HiSeq 2000 and Illumina Genome Analyzer IIx*
Data format	*DNase-seq: BROADPEAK, NARROWPEAK, BIGWIG; ChIP-seq: BED, BIGWIG; RNA-seq: BIGWIG, TXT*
Experimental factors	*Adult tissues (brain, spleen, ovary) in which the Xi is from spretus, and Patski fibroblast cell line in which the Xi is from BL6.*
Experimental features	*RNA-seq to identify genes escaping X chromosome inactivation in a tissue-specific manner, and DNase-seq and ChIP-seq to identify molecular mechanisms involved in escape.*
Consent	*NA*
Sample source location	*Riken Institute, Japan and Jackson Laboratory, USA*

## Direct link to deposited data

The Patski cell DNase-seq and RNA-seq data is available through GEO series GSM970866 and GSM1014171, respectively, and ENCODE data portals:

https://www.encodeproject.org

http://www.mouseencode.org

The ChIP-seq data for RNA polymerase PolII-S5p occupancy in brain and Patski cells are available under GSE44255:

http://www.ncbi.nlm.nih.gov/geo/query/acc.cgi?acc=GSE44255

The RNA-seq data for tissues and ChIP-seq data for CTCF binding in brain and Patski cells are available under GSE59779:

http://www.ncbi.nlm.nih.gov/geo/query/acc.cgi?acc=GSE59779

Individual dataset descriptions can be found in Table 1.

## 1 Value of the data

• The data describe tissue specific escape gene profiles, discovery of which could lead to insights into the contribution of escape genes in sex chromosome aneuploidy such as Turner syndrome and in sex differences in general.
• The data show that tissue specific escape genes have tissue specific functions hinting at potential roles for X-linked bi-allelic expression.
• Analysis of chromatin architecture at escape genes will lead to a better understanding of the mechanisms underlying bi-allelic X-linked gene expression.

## 2 Data, experimental design, materials and methods

Random inactivation of one of the X chromosomes in mammalian females takes place during early development and is associated with *Xist* coating, accumulation of repressive histone modifications and a specific alteration of chromatin architecture. A few genes escape random X inactivation and remain bi-allelicly expressed throughout the life of the organism. This data is associated with the research article focused on identifying escape genes in multiple tissue types in vivo, and on investigating a mechanism that contributes to expression from the inactive X using a novel hybrid mouse model.

### 2.1 in vivo mouse model

**Fig. 1:**
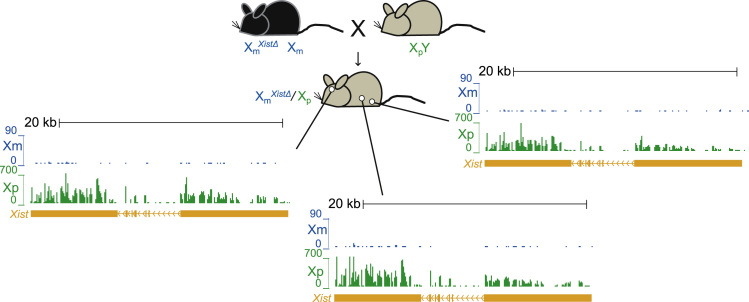
F1 mouse model with skewed X inactivation and identifiable alleles**.** C57BL6 females with a *Xist* deletion [2] were mated with wild-derived *Mus spretus* males. In the resulting F1 females the BL6 maternal X chromosome that carries the *Xist* mutation (Xm^*Xist*Δ^) cannot become inactivated, leading to complete skewing of X inactivation where the paternal *spretus* X chromosome (Xp) is inactivated in all tissues. Shown are UCSC genome browser tracks of mRNA SNP read distribution profiles on the active Xm (blue) and the inactive Xp (green) in brain, spleen and ovary at the *Xist* gene. Note that *Xist* is expressed from the *spretus* Xp in all tissues analyzed.

Increased *Xist* expression during development is necessary for normal X inactivation. Several regions of the *Xist* gene contain repetitive elements which are conserved between mouse and humans. The proximal A-repeat has been shown to be necessary for *Xist* expression and thus, X inactivation [2]. In order to derive a mouse model with skewed X inactivation, we took advantage of a previously described mouse model in which the proximal A-repeat of *Xist* (*Xist*^Δ^) was deleted (B6.Cg-Xist<tm5Sado>, RIKEN [2]. We bred heterozygous C57BL/6 (BL6) females with the mutant *Xist* (*Xist*^Δ/+^) to wild *Mus spretus* males. The mutant maternal X (X_m_) fails to inactivate, thus the resulting F1 females that inherited the mutant X_m_ have identifiable alleles and completely skewed X inactivation in which the Xi is always the paternal *spretus* X (X_p_) chromosome (Fig. 1).

Mice were genotyped to verify mutant status using primers previously described [2]. F1 females heterozygous for the *Xist* mutation were euthanized at 8 wks of age. Chromatin, DNA and RNA from whole brain, spleen and ovaries from mutant F1 females (X_m_^*Xist*Δ^/X_p_) were isolated according to the protocols described below. Skewing of X inactivation was verified by Sanger sequencing as previously described [1].

### 2.2 in vitro cell culture

Patski cells were derived from the kidney of an 18dpc F1 female embryo from a cross between a *M. spretus* male and a C57BL/6 female with an *Hprt* mutation. Briefly, embryonic kidney cells were selected in media containing hypoxanthine, aminopterin and thymidine (HAT). Only cells with a functional *Hprt* gene are able to survive in HAT media. Thus, only F1 embryonic kidney cells that contained a Xa from *spretus* and a Xi with the mutant allele (BL6^*Hprt−*^) survived. X inactivation in the selected cells (Patski cells) was completely skewed wherein the *spretus* X was always active [3]. After selection, the Patski cell line was immortalized and was cultured in Dulbecco׳s Modified Eagle׳s Medium (DMEM) with 10% FBS and 1% penicillin/streptomycin at 37 °C in 5% CO_2_.

### 2.3 RNA extraction and RNA-seq

Total RNA was extracted from homogenized *Xist*^Δ/+^ hybrid tissues or from Patski cell pellets using Qiagen׳s RNeasy mini kit (Qiagen #74104). For the RNA-seq library of Patski cells, the Illumina mRNA-seq preparation kit was used in our previous study [4]. For the RNA-seq libraries of mouse tissues, we used the Illumina TruSeq RNA preparation kit (Illumina #RS-930-2001) with some slight modifications. For mRNA isolation, 0.5–4 µg of total RNA was diluted into a total volume of 30 µl with nuclease free water then combined with 30 µl of oligo dT magnetic beads. After incubation at 65 °C for 5 min, beads were washed with 120 µl washing buffer and eluted with 30 µl elution buffer. Following a second round of mRNA selection, mRNA was eluted and fragmented at 94 °C for 8 min. First-strand cDNA synthesis, second-strand cDNA synthesis and end repair were performed according to the TruSeq instructions except that all clean up steps were done using a Qiagen mini-elute columns (Qiagen #28004). A-tailing was done according to the TruSeq protocol incorporating the version 2 changes and substituting AMpure XP beads with Qiagen mini-elute columns as described above. Further processing, including adapter ligation and fragment enrichment were done according to the TruSeq protocol except that clean up steps were done with Qiagen columns. Libraries were sequenced using an Illumina Genome Analyzer IIx generating 36 bp single-end reads with analyses described below.

### 2.4 Chromatin immunoprecipitation and ChIP-seq

In order to study the chromatin characteristics at escape genes in F1 tissues and Patski cells, we carried out ChIP using specific antibodies for CTCF and RNA PolII-S5p, respectively. F1 tissues were collected and homogenized in a pre-chilled 7 ml glass homogenizer in 5 ml PBS containing 0.1 mM phenylmethylsulfonyl fluoride (PMSF) on ice. The tissue homogenates were transferred to a 15 ml tube and pelleted followed by re-suspension in 10 ml PBS. Samples were cross-linked in 1% formaldehyde for 10 min at room temperature and 125 mM final concentration of glycine was added to quench cross-linking. After washing with ice cold PBS containing 0.1 mM PMSF, cross-linked cells were re-suspended in immuneprecipitation (IP) buffer [5] containing 0.1 mM PMSF and separated into 1 ml aliquots which were either stored at −80 °C or lysed in IP buffer for 10 min on ice. For PolII-S5p ChIP, IP buffer per 1 ml was also supplemented with the following phosphatase inhibitors: 10 µl of 1 M β-glycerophosphate, and 10 µl of 1 M NaF, 1 µl of 100 mM Na_3_VO_4_. Shearing of chromatin was accomplished by sonication (10 rounds of fifteen 1 s pulses at power 6; 2 min rest on ice between rounds) using a Misonix 3000 sonicator. Chromatin concentration was measured using a Nanodrop followed by pre-clearing with 100 µl protein A beads (GE Healthcare Life Sciences) for 1 h at 4 °C. All of the steps described above were also done for Patski cells with the exception of the initial homogenization.

For IP, 100 µg of cross-linked pre-cleared chromatin was combined with either 10 µg anti-CTCF (Millipore, 07-729) or 5 µg PolII-S5p (Abcam, ab5131) antibodies and incubated in 1 ml IP buffer over night at 4 °C with rotation. Ten percent of cross-linked pre-cleared chromatin (10 µg) was saved as the input control. Mock ChIP (no antibody control) was also simultaneously performed. For collection of DNA-protein-antibody complexes, immunoprecipitated chromatin was incubated with 100 µl protein A beads at 4 °C for 2 h with rotation followed by washes in buffers with varying concentrations of salts (low salt: 0.1% SDS, 1% Triton-X-100, 20 mM EDTA, 20 mM Tris–HCl, 150 mM NaCl; high salt: 0.1% SDS, 1% Triton-X-100, 20 mM EDTA, 20 mM Tris–HCl, 500 mM NaCl; LiCl buffer: 250 mM LiCl, 1% NP-40, 1% deoxycholic acid, 1mM EDTA, 10 mM Tris pH 8.1) followed by two TE washes to remove any residual salt. All wash steps were done at 4 °C. Complexes were eluted from the beads in elution buffer (1% SDS; 100 mM NaHCO_3_) 2 times for 20 min each at room temperature. To reverse the cross-links, eluted complexes were incubated in elution buffer supplemented with 200 mM NaCl overnight at 65 °C. ChIP׳d DNA was purified using QIAquick PCR purification columns (Qiagen #28106). An aliquot of ChIP and input DNA subjected to PCR for the housekeeping gene β-actin to confirm specificity of the pull-down compared to the no-antibody mock ChIP control.

Purified DNA from CTCF or PolII-S5p ChIP experiments in brain as well as PolII-S5p ChIP experiments in Patski cells were used for library preparation according to the Illumina TruSeq ChIP preparation protocol, with the exception that all AMPure XP bead purification steps were replaced by Qiagen mini-elute columns. Briefly, ChIP DNA was repaired to generate blunt ends followed by the addition of an “A” nucleotide to each end. Adapters were ligated and 300–650 bp size fragments were selected in a 1.5% low-melting agarose gel followed by column purification. After PCR enrichment of purified fragments, completed libraries were sequenced on an Illumina HiSeq 2000 machine. Purified DNA from CTCF ChIP experiments in Patski cells was used for library preparation according to the Illumina genomic DNA preparation protocol and sequenced on an Illumina Genome Analyzer IIx. Sequenced libraries were analyzed as described below.

### 2.5 Mapping and allele-assignment of sequencing reads

To identify reads that map to each parental genome of the F1 mice, we first assembled a "pseudo-*spretus*" genome by substituting known SNPs of *spretus* into the BL6 NCBIv37/mm9 reference genome. *Spretus* SNPs were obtained from the Sanger Institute (SNP database Nov/2011 version) and from in house analysis [4]. In total, we collected 1,532,011 heterozygous SNPs on the X chromosome and 31,062 of them were located in exonic regions. A more detailed breakdown of SNP location can be found in Table 2.

Reads from genomic DNA sequencing, ChIP-seq, and DNaseI-seq experiments were mapped separately to the BL6 reference sequence (mm9) and to the pseudo-*spretus* genome using BWA/v0.5.9 [6] with default parameters. RNA-seq reads from tissue samples were mapped to both the BL6 and the pseudo-*spretus* genomes and transcriptomes using Tophat/v2.0.2 [7] with default parameters. We segregated all high-quality (MAPQ≥30) uniquely mapped reads into three categories: (1) BL6-SNP reads containing only BL6-specific SNP(s); (2) *spretus*-SNP reads containing only *spretus*-specific SNP(s); (3) allele-uncertain reads, that is, reads that do not contain valid SNPs (Table 3). We refer to both BL6-SNP reads and *spretus*-SNP reads as "allele-specific reads".

### 2.6 RNA-seq analysis and identification of escape genes

We calculated diploid gene expression based on all high-quality uniquely mapped reads using cufflinks/v2.0.2 [8] to determine the gene-level RPKM (reads per kb of exon length per million mapped reads) expression values. In addition, we defined SNP-based haploid gene expression from alleles on the Xi or the Xa (Xi-SRPM or Xa-SRPM) to be allele-specific SNP-containing exonic reads per 10 million high-MAPQ uniquely mapped reads.

To identify escape genes and estimate the statistical confidence of escape probability of X-linked genes, we developed the following binomial model. For each gene i on chromosome X, let the number of allele-specific RNA-seq reads mapped to the inactive/active chromosomes be $n_{i0}$ and $n_{i1}$, respectively, and let ${n_{i}=n_{i0}+n}_{i1}$. We model $n_{i0}$ by a binomial distribution:

$$n_{i0}\sim \mathrm{Binomial}(n_{i},p_{i}),$$

where $p_{i}$ indicates the expected proportion of reads from the Xi. The estimate of the binomial proportion is $\hat{p_{i}}=\frac{n_{i0}}{n_{i}}$. Let $z_{\frac{\alpha }{2}}$ be the $100{\left(1-\frac{\alpha }{2}\right)}^{\mathrm{th}}$ percentile of *N*(0,1). The confidence interval of each $\hat{p_{i}}$ is $\hat{p_{i}}\pm z_{\frac{\alpha }{2}}\sqrt{\frac{\hat{p_{i}}\left(1-\hat{p_{i}}\right)}{n_{i}}}$.

Inclusion of a mapping bias correction was necessary since our model utilizes distantly related mouse species and prevents analysis of reciprocal crosses [1]. To incorporate the mapping biases toward the BL6 genome over the pseudo-*spretus* genome into the above model, we define the mapping bias ratio $r_{m}$ for each RNA-seq experiment to be $r_{m}=\frac{N_{A0}}{N_{A1}}$, where $N_{A0}$ and $N_{A1}$ are the number of allele-specific autosomal reads in the "inactive X containing" genome and the "active X containing" genome, respectively. Considering the mapping biases, the corrected estimate of $p_{i}$ is $\bar{p_{i}}=\frac{n_{i0}}{\left(n_{i0}+r_{m}n_{i1}\right)}=\frac{\hat{p_{i}}}{\left(\hat{p_{i}}+r_{m}\left(1-\hat{p_{i}}\right)\right)}$. The upper and lower confidence limits are corrected accordingly.

**Fig. 2:**
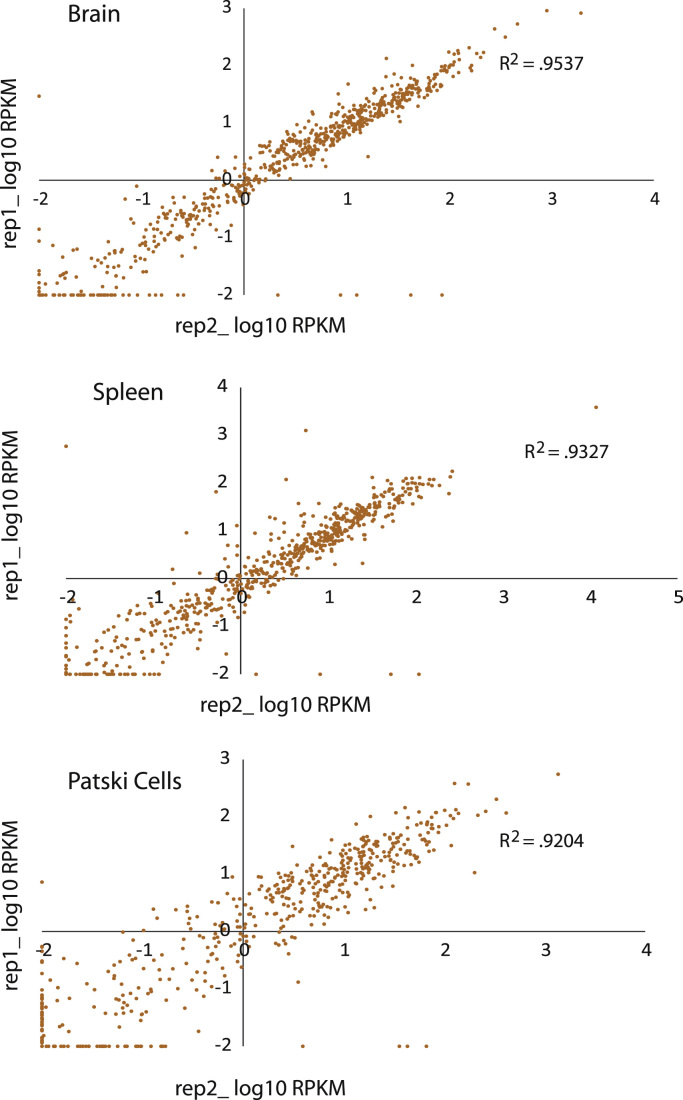
Scatter plots showing data reproducibility of RNA-seq in tissues and cells**.** Plots represent expression levels from the X chromosome in replicate 1 (rep1) versus expression levels from the X chromosome in replicate 2 (rep2).

For each RNA-seq experiment, we defined an X-linked gene as an "*escape*" gene using the following three criteria: (1) the 99% lower confidence limit (α=0.01) of the escape probability $\bar{p_{i}}$ was greater than zero, (2) the diploid gene expression measured by RPKM was ≥1, indicating that the gene was expressed, and (3) the normalized Xi-SRPM was ≥2 but <5 (low-level escape) or ≥5 (high-level escape). Biological replicates of RNA-seq experiments were analyzed separately. The Xi-SRPM values are highly correlated between biological replicates (*R*^2^>0.9) (Fig. 2).

### 2.7 ChIP-seq analysis

For CTCF and PolII-S5p ChIP-seq data, we first used all high-MAPQ uniquely mapped reads to identify enriched peak regions in the diploid genome. Two independent peak calling programs were applied, CisGenome (FDR cutoff 10^−5^) [9], [10] and MACS/v1.4 (*p*-value cutoff 10^−5^) [11]. We defined significantly enriched peak regions as those identified by both peak callers (Table 4).

Next we identified allele-specific ChIP-seq peaks by the following binomial test. In each diploid ChIP-seq peak region i, we assumed that the numbers of BL6-SNP reads ($n_{i,\mathrm{bl}}$) and *spretus*-SNP reads ($n_{i,\mathrm{sp}}$) within the peak follow a binomial distribution, i.e.,

$$n_{i,\mathrm{bl}}\sim \text{Binomial}\left(n_{i},p_{i}\right),$$

where $n_{i}=n_{i,\mathrm{bl}}+n_{i,\mathrm{sp}}$ is the sum of BL6-SNP reads and *spretus*-SNP reads in peak region i, and $p_{i}$ is the binomial parameter. Since the X chromosome behaves differently from autosomes due to skewed XCI in our systems, we estimated the X chromosome allelic background using all SNP reads in the identified diploid peak regions on the X only. That is, for peaks on the X chromosome,

$$p_{i}=p_{X}=\frac{N_{X,\mathrm{bl}}}{\left(N_{X,\mathrm{bl}}+N_{X,\mathrm{sp}}\right)},$$

in which $N_{X,\mathrm{bl}}$ and $N_{X,\mathrm{sp}}$ are the total number of BL6-SNP and *spretus*-SNP reads in X peaks, respectively. Finally, BL6-preferred ChIP-seq peaks were defined as those that contain significantly more BL6-SNP reads (upper-tail binomial test, *p*-value<0.05), while *spretus*-preferred ChIP-seq peaks were identified using the lower-tail binomial test (*p*-value<0.05), and both-preferred ChIP-seq peaks were those peaks that were not significant in the two above tests (*p*-value≥0.25) (Table 4). In addition, we required the allele-assessable peaks have a minimal SNP read coverage of one allele-specific read (BL6-SNP and *spretus*-SNP reads) per 10 million mapped reads.

## Figures and Tables

**Table 1:** Datasets and corresponding URLs.

*Sample*	*Replicate*	*Feature*	*Upload URLs*
Brain	1	RNA-seq	http://www.ncbi.nlm.nih.gov/geo/query/acc.cgi?acc=GSM1446324
Brain	2	RNA-seq	http://www.ncbi.nlm.nih.gov/geo/query/acc.cgi?acc=GSM1446325
Spleen	1	RNA-seq	http://www.ncbi.nlm.nih.gov/geo/query/acc.cgi?acc=GSM1446326
Spleen	2	RNA-seq	http://www.ncbi.nlm.nih.gov/geo/query/acc.cgi?acc=GSM1446327
Ovary	1	RNA-seq	http://www.ncbi.nlm.nih.gov/geo/query/acc.cgi?acc=GSM1446328
Patski	1^a^	RNA-seq	https://www.encodeproject.org/experiments/ENCSR000CLX/
Patski	2^a^	RNA-seq	http://www.ncbi.nlm.nih.gov/sra?term=SRA010053
Brain	1	CTCF ChIP-seq	http://www.ncbi.nlm.nih.gov/geo/query/acc.cgi?acc=GSM1446329
Brain	1	PolII ChIP-seq	http://www.ncbi.nlm.nih.gov/geo/query/acc.cgi?acc=GSM1081374
Patski	1	CTCF ChIP-seq	http://www.ncbi.nlm.nih.gov/geo/query/acc.cgi?acc=GSM1446330
Patski	1	PolII ChIP-seq	http://www.ncbi.nlm.nih.gov/geo/query/acc.cgi?acc=GSM1446331
Patski	1^b^	Dnase-seq	https://www.encodeproject.org/experiments/ENCSR000CNT/
Patski	2^b^	Dnase-seq	https://www.encodeproject.org/experiments/ENCSR000CNT/

a Patski cell RNA-seq, data deposited by ENCODE (1) and data from [4] (2) re-analyzed using our new binomial model.

b Analysis was done using two replicates of DNase-seq data deposited by ENCODE.

**Table 2:** SNP location summary.

*Location*	*Total SNPs*^a^	*Number of genes with exonic SNPs*^b^
X chromosome	1,532,011 (4.3%)	1099 (73.6%)
Autosomes	33,909,724 (95.7%)	27,010 (93.1%)
Both	35,441,735 (100%)	28,109 (92.1%)

a Total number of SNPs at the corresponding chromosomal locations (X, autosomes or both).

b Number of genes with exons containing SNPs at the corresponding chromosomal locations (X, autosomes or both).

**Table 3:** Mapped reads summary.

*Sample*	*Experiment*	*Replicate*	*Length* (bp)	*Raw reads*	*% unique*^a^	*% BL6*^b^	*% spretus*^b^	*% uncertain*^b^	*Ratio*^c^
Brain	RNA-seq	1	36	104,241,363	80	11	10	78	1.1
Brain	RNA-seq	2	36	36,430,212	86	12	11	77	1.1
Spleen	RNA-seq	1	36	88,842,032	73	12	11	77	1.07
Spleen	RNA-seq	2	36	32,538,760	72	12	11	76	1.07
Ovary	RNA-seq	1	36	78,210,224	77	11	11	78	1.06
Patski	RNA-seq	1	36	33,784,231	79	11	10	79	1.04
Patski	RNA-seq	2	36	27,196,142	67	10	10	80	1.04
Brain	CTCF ChIP-seq	1	100	552,106,670	51	43	30	27	1.43
Patski	CTCF ChIP-seq	1	36	87,390,982	56	22	20	58	1.13
Brain	PolII ChIP-seq	1	100	301,496,879	44	40	32	28	1.26
Patski	PolII ChIP-seq	1	36	132,743,111	48	22	20	58	1.11
Patski	Dnase-seq	1	36	36,361,296	65	18	17	65	1.09
Patski	Dnase-seq	2	36	38,191,194	68	19	18	63	1.09

a Percent of uniquely mapped reads calculated over the number of raw reads.

b Percents of BL6, *spretus* and allele-uncertain reads calculated over the number of high-quality uniquely mapped reads. % BL6 and % *spretus* reads represent reads containing only BL6 or *spretus* SNPs, respectively. % uncertain reads represents reads that contained no valid SNPs.

c Ratios were calculated as BL6-SNP reads/ *spretus*-SNP reads.

**Table 4:** ChIP-seq peak numbers.

*Sample*	*Protein*	*# Total peaks*^a^	*# Total X-linked peaks*^b^	*# Xa-preferred*^c^	*# Xi-preferred*^c^	*# Both-preferred*^c^
Brain	CTCF	119,860	2508	1060	211	365
Patski	CTCF	27,810	606	126	86	160
Brain	PolIIS5p	58,576	792	133	37	267
Patski	PolIIS5p	45,714	550	180	80	86

a Number of total peaks identified.

b Number of peaks present on the X chromosomes.

c Number of peaks preferred on the Xa, Xi or both.
